# Cecal Duplication Cyst: A New Surgical Intervention

**DOI:** 10.7759/cureus.44613

**Published:** 2023-09-03

**Authors:** Adel A Alfayez, Zafer Skef

**Affiliations:** 1 Department of Pediatric Surgery, Prince Sultan Military Medical City, Riyadh, SAU; 2 Division of Pediatric Surgery, Department of Surgery, Security Forces Hospital, Riyadh, SAU

**Keywords:** cecal duplication cyst, case report, bowel obstruction, bilious vomiting, pediatric age group, caecal duplication cyst

## Abstract

This case report of an infant details a rare occurrence of a cecal duplication cyst causing bowel obstruction. It was successfully treated through an extra mucosal enucleation. The patient presented at 41 days of life, with two days picture of abdominal distension and recurrent non-bilious vomiting. The infant improved initially, but subsequently, he developed bilious vomiting. Further investigations revealed a suspected ileocolic intussusception and small bowel obstruction. Surgical exploration revealed a cecal duplication cyst. The cyst was enucleated, and closure of the seromuscular defect was done with an appendectomy. The patient had a smooth recovery postoperatively. Histopathology confirmed the presence of a duplication cyst with benign ectopic gastric tissue negative for malignancy. The patient was discharged without any complications.

## Introduction

Cecal duplication cysts (CDCs) are an uncommon congenital abnormality of the gastrointestinal tract that can result in intestinal obstruction [1]. These enteric duplication cysts (EDCs) can manifest at various locations throughout the gastrointestinal tract, with the ileum and stomach being the most frequent sites [1]. However, they can also occur in the retroperitoneum, although isolated duplication cysts in this region are infrequent [1]. EDCs are categorized as foregut, midgut, or hindgut, depending on their originating site. A cyst is classified as an EDC when it possesses a smooth muscle layer, a gastrointestinal type of epithelial lining, and some connection to the gastrointestinal tract [2].

According to estimates, CDCs account for a mere 0.4% of all gastrointestinal duplications [3]. Typically, CDCs manifest as either cystic or tubular structures [4], with cystic duplications being particularly uncommon [5]. Additionally, it is worth noting that these cysts have the potential to harbor carcinoid tumors [6].

The clinical manifestations of duplication cysts exhibit a significant degree of variability, contingent upon the specific type, size, and location of the lesion. Generally, symptoms arise within the first two years of life, prompting the necessity for radiological assessment to facilitate an accurate diagnosis [7]. These symptoms encompass abdominal distention, abdominal pain, obstruction, bleeding, compromised respiration, and the presence of a painless mass [8,9]. Additionally, the majority of patients typically display signs indicative of sub-acute intestinal obstruction, whereas the acute onset of symptoms generally signifies complications such as intracystic hemorrhage, perforation, or infection [10]. In the case of CDCs, common symptoms include bilious vomiting and abdominal distention. Furthermore, CDCs may also exhibit symptoms resembling those of intussusception [11]. Consequently, it is imperative to possess a comprehensive understanding of the underlying anatomical and embryological aspects of the cecum to accurately diagnose and manage this condition.

In this article, our objective is to present a case study of an infrequent occurrence: a duplication cyst in the cecum. The management of this particular case involved a surgical procedure known as enucleation, which yielded favorable results without any complications. Additionally, we encountered a diagnostic dilemma, highlighting the importance of raising awareness within the healthcare community when faced with similar cases.

## Case presentation

**Figure 1:**
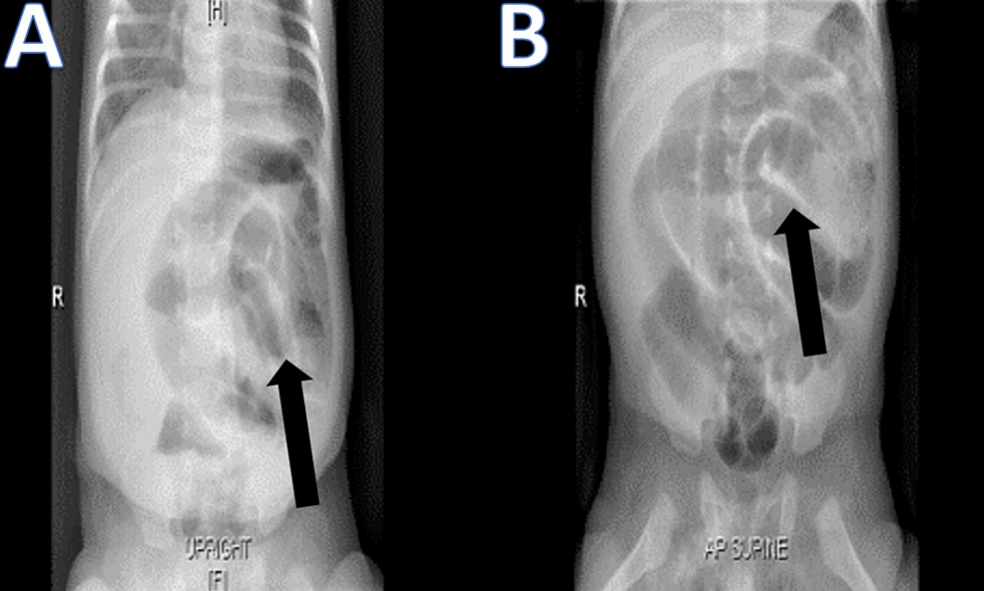
X-ray of the abdomen A: Erect X-ray of the abdomen showed thickened, dilated bowel loops (arrow) with no air-fluid levels. B: Supine X-ray of the abdomen revealed thickened, dilated bowel loops (arrow) with no air-fluid levels.

**Figure 2:**
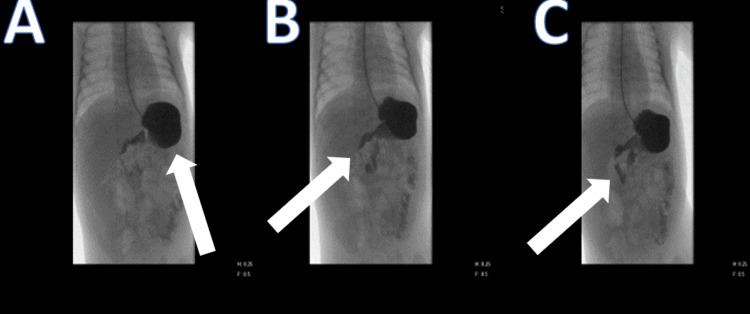
Upper gastrointestinal study A: Image showing the contrast filling the stomach. B: The duodenal C loop, which appears unremarkable. C: Normal transit of contrast noted from the stomach into the small bowel loops.

**Figure 3:**
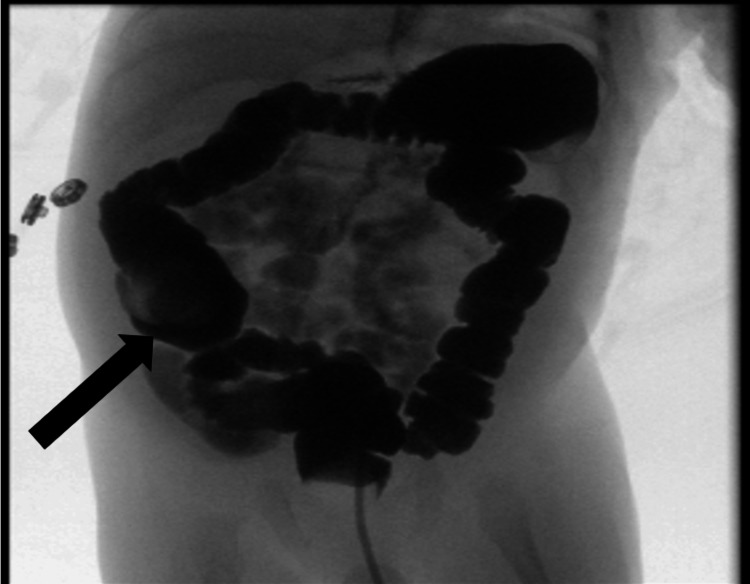
Lower gastrointestinal study Large bowel lobes appear unremarkable with no fluoroscopic evidence of malrotation or radiological filling defects (arrow).

**Figure 4:**
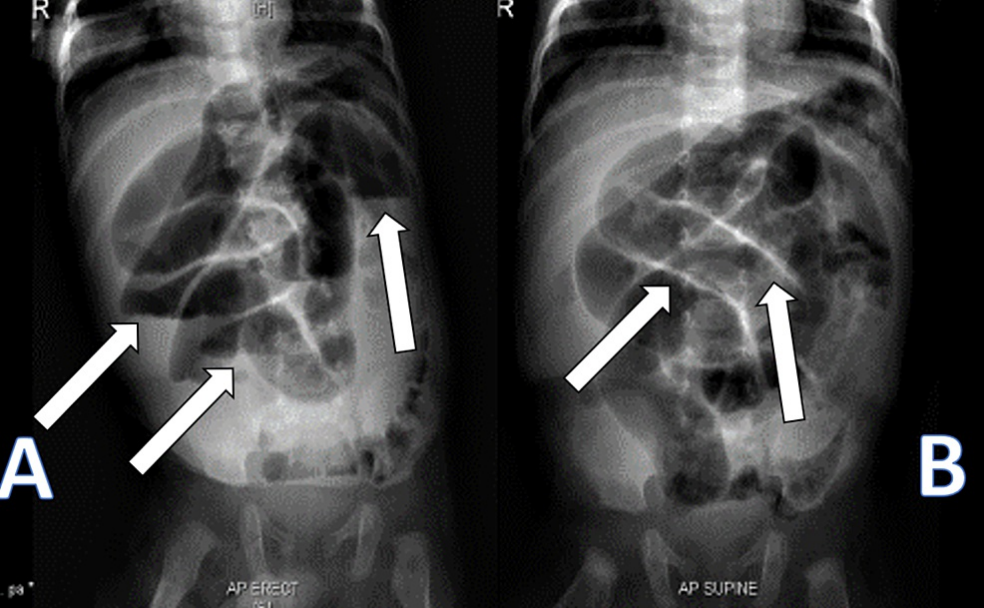
X-ray of the abdomen A: Erect X-ray of the abdomen demonstrates multiple air-fluid levels. B: Supine X-ray of the abdomen showed thickened, dilated bowel loops.

**Figure 5:**
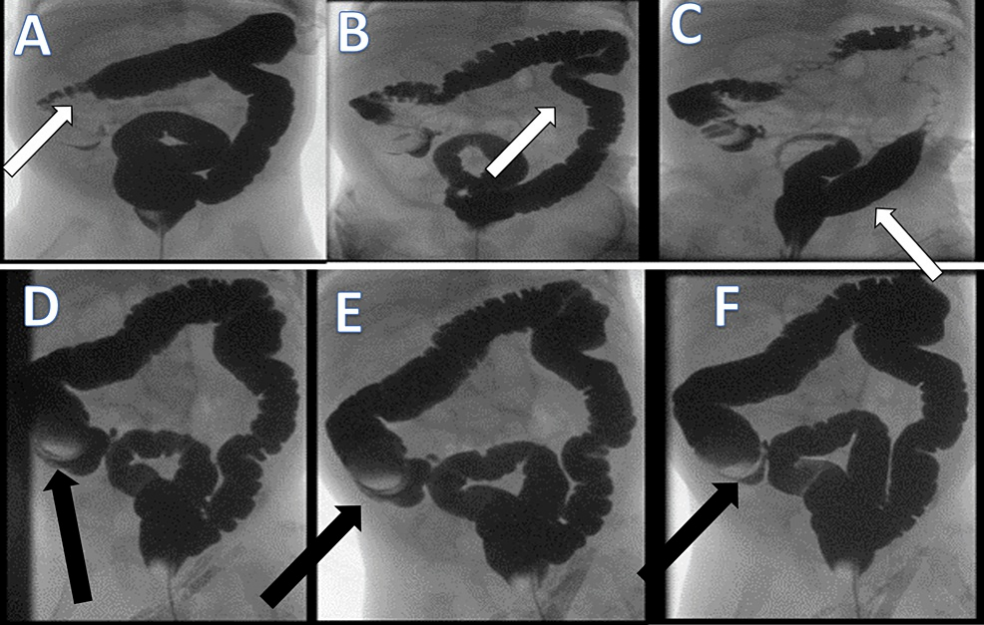
Lower gastrointestinal study A-C: Lower study contrast passed smoothly (arrows) to the ileocecal area, with suggestive findings. D-F: Images demonstrating suggestive finding with questionable ileocecal intussusception and filling defect at the ileocecal area, and a 2.5 cm mass with filling defect at the ileocecal area (arrows).

**Figure 6:**
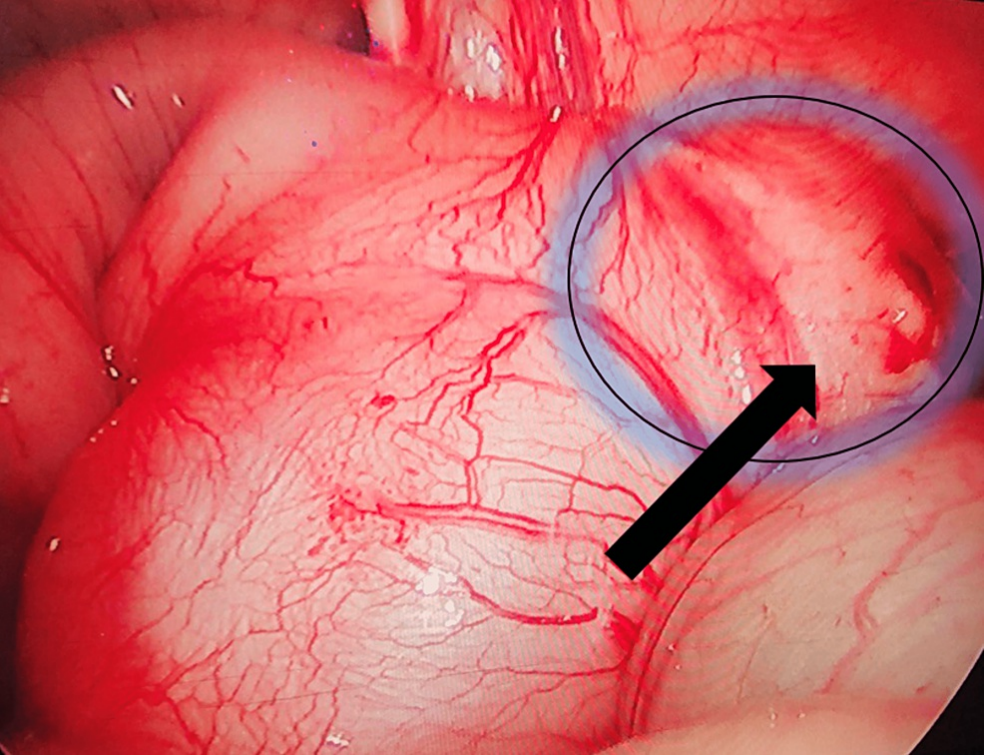
Intraoperative finding During laparoscopic exploration, a suspicious cystic lesion was seen, which was a duplication cyst (black arrow).

**Figure 7:**
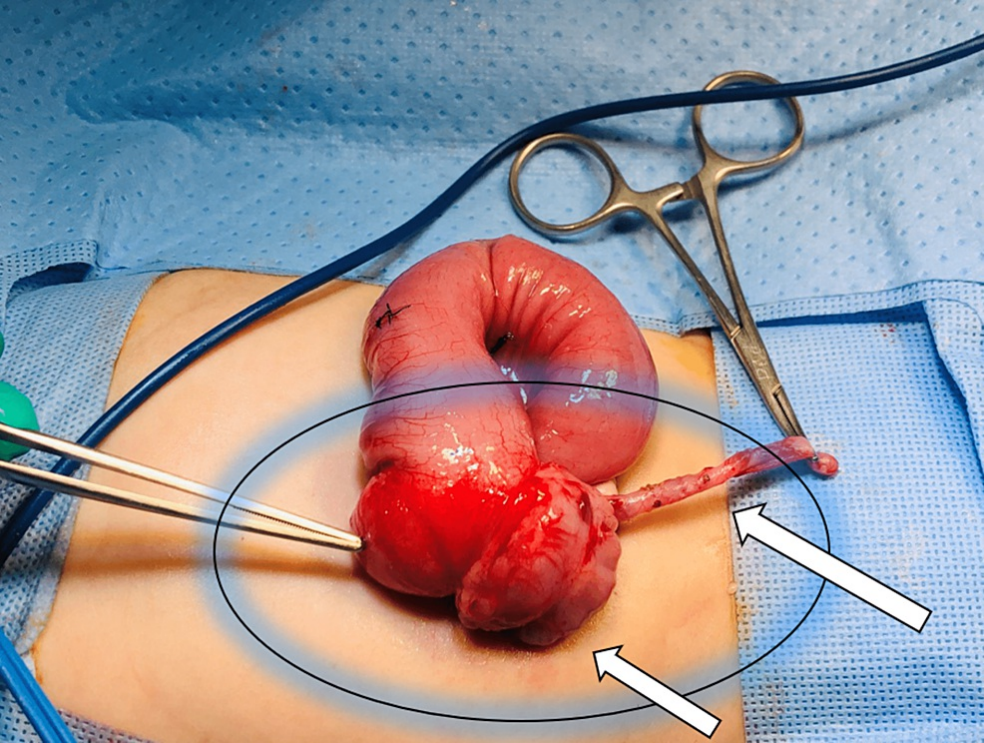
Intraoperative finding The laparoscopic approach was converted to an open procedure. Intraoperative findings showing the surrounding terminal ileum, appendix (long arrow), and cecum (short arrow), along with the duplication cyst (pointed DeBakey forceps).

This is a case of a 41-day-old infant boy with no known medical conditions. The mother had an uneventful antenatal period. The baby had regular prenatal check-ups and passed meconium within the first 24 hours. The baby was brought to the emergency department with a history of repeated vomiting, which later turned into bilious over the past two days. The infant had mild abdominal distension and regular bowel movements. There were no other significant symptoms. An abdominal X-ray (Figure 1) showed dilated bowel loops. Abdominal ultrasonography was unremarkable, with no evidence of intussusception. An upper gastrointestinal series, followed by a lower gastrointestinal contrast study, did not reveal any significant findings (Figures 2, 3). The baby was admitted for close monitoring for two days, during which the baby tolerated feeding well and had regular bowel movements. He was discharged with clear instructions and a scheduled follow-up. Seven days later, the baby developed abdominal distension and recurrent bilious vomiting for one day. A repeat abdominal X-ray (Figure 4) showed prominent distended bowel loops. The lower gastrointestinal contrast study suggested distal bowel obstruction caused by ileocecal intussusception and the presence of a 2.5 cm mass or filling defect, as depicted in Figure 5. The initial decision was to proceed with laparoscopic exploration, but it was ultimately converted to laparotomy (Figures 6, 7).

**Figure 8:**
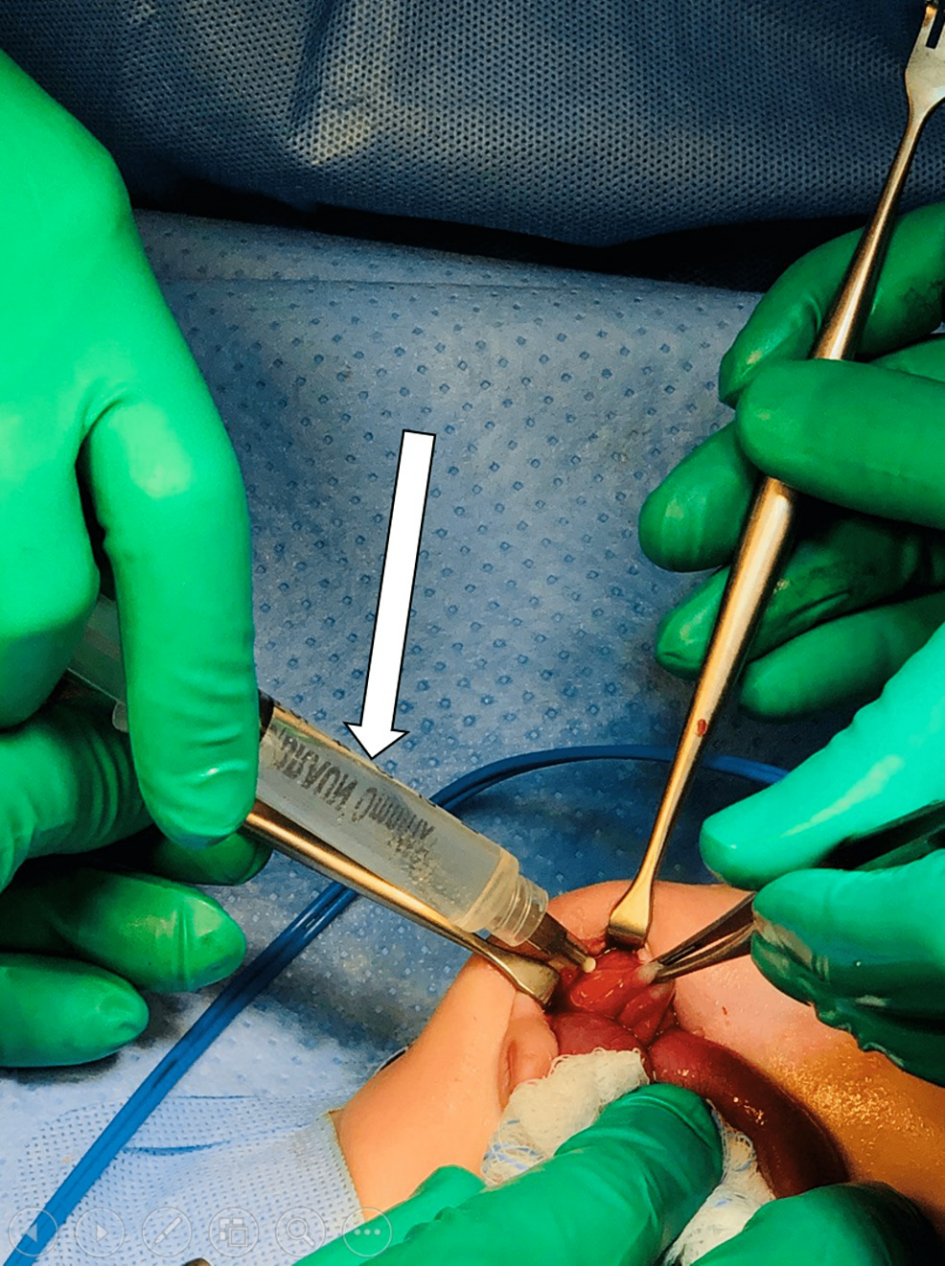
Intraoperative findings The laparoscopic approach was converted to an open procedure, with clear aspirated cystic fluid of 3-5 ml as demonstrated (white arrow).

**Figure 9:**
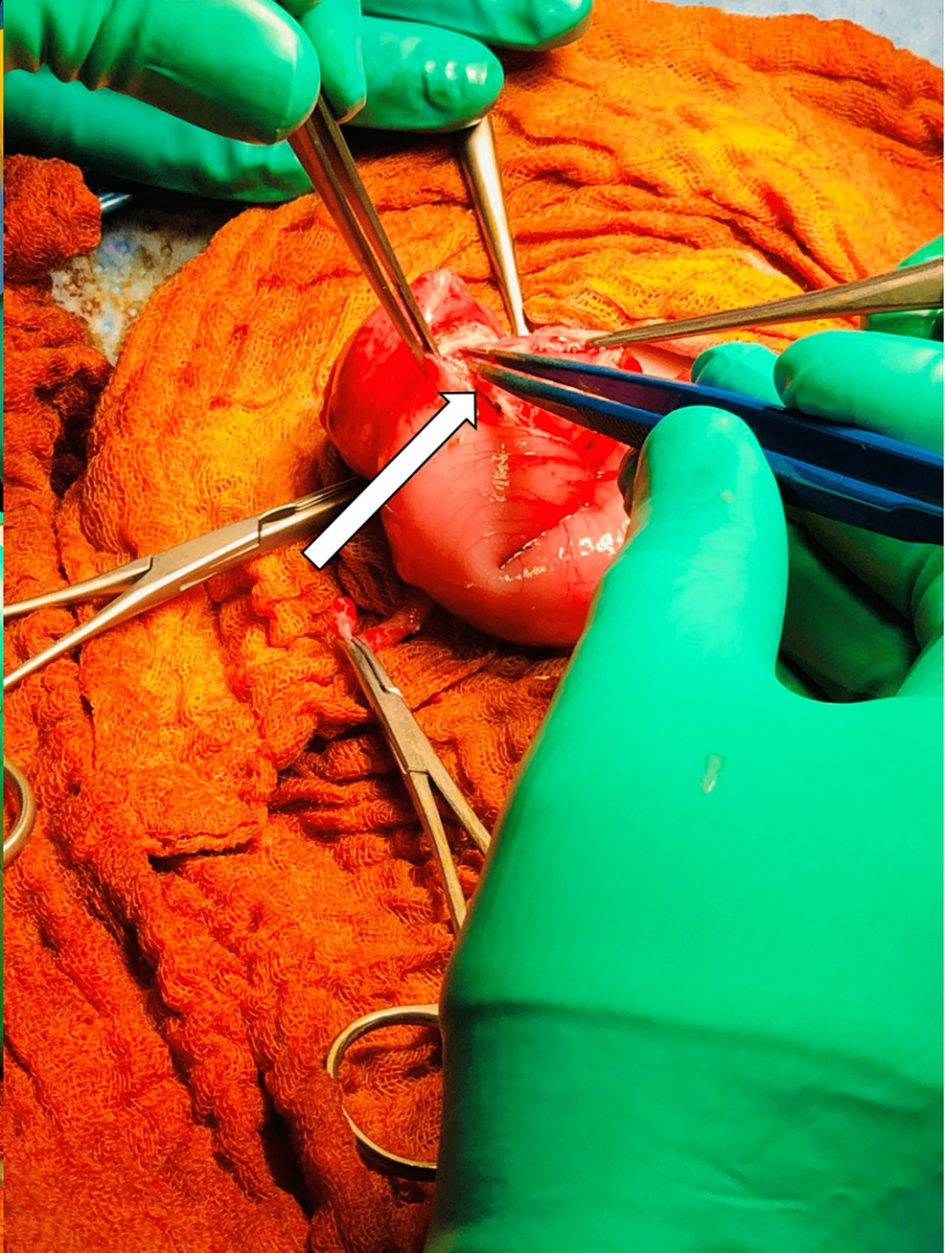
Intraoperative findings Image showing the cystic lesion and steps of submucosal enucleation using bipolar diathermy (arrow).

**Figure 10:**
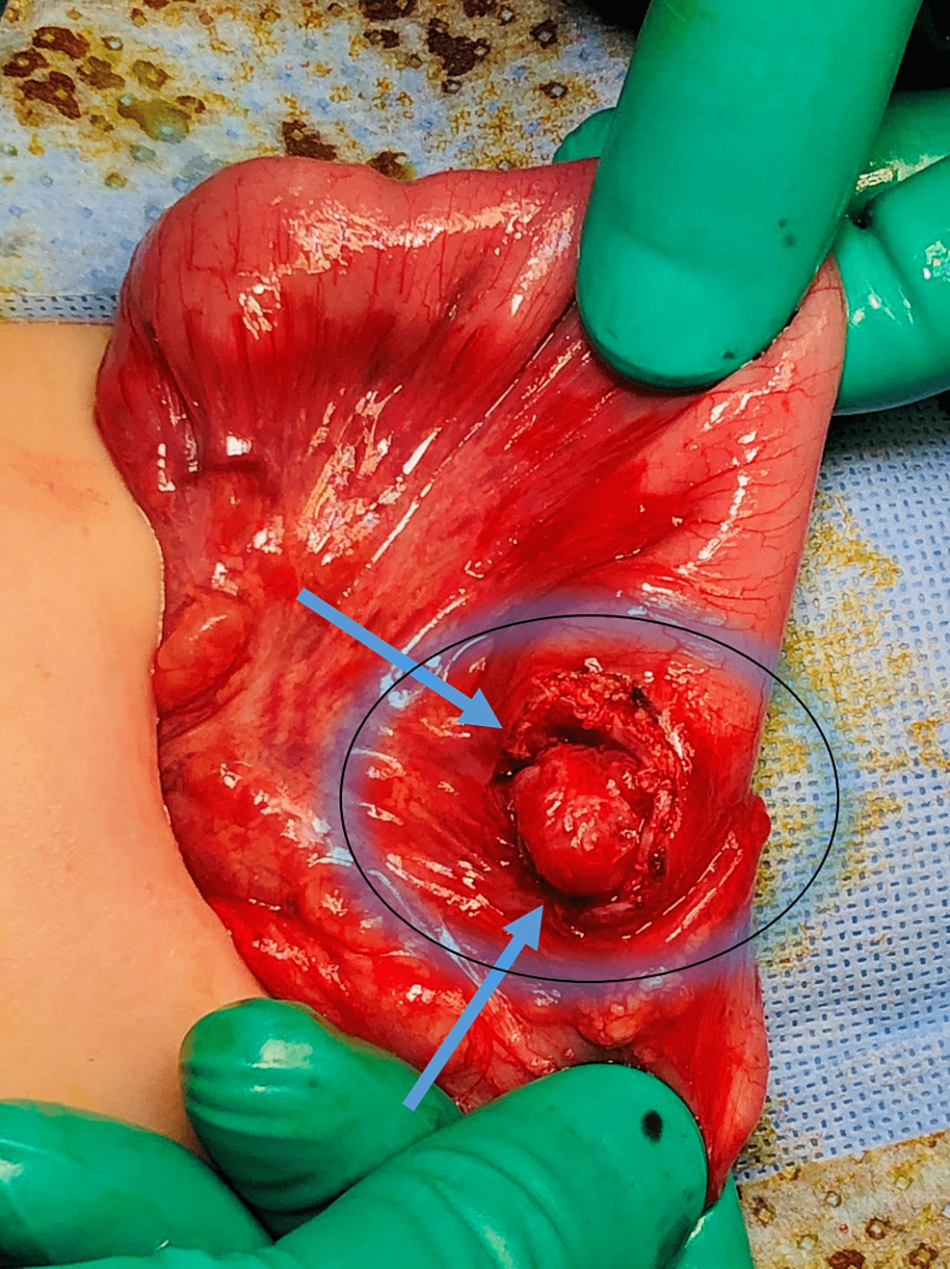
Intraoperative finding Post submucosal enucleation with intact mucosal layer edges (arrows).

**Figure 11:**
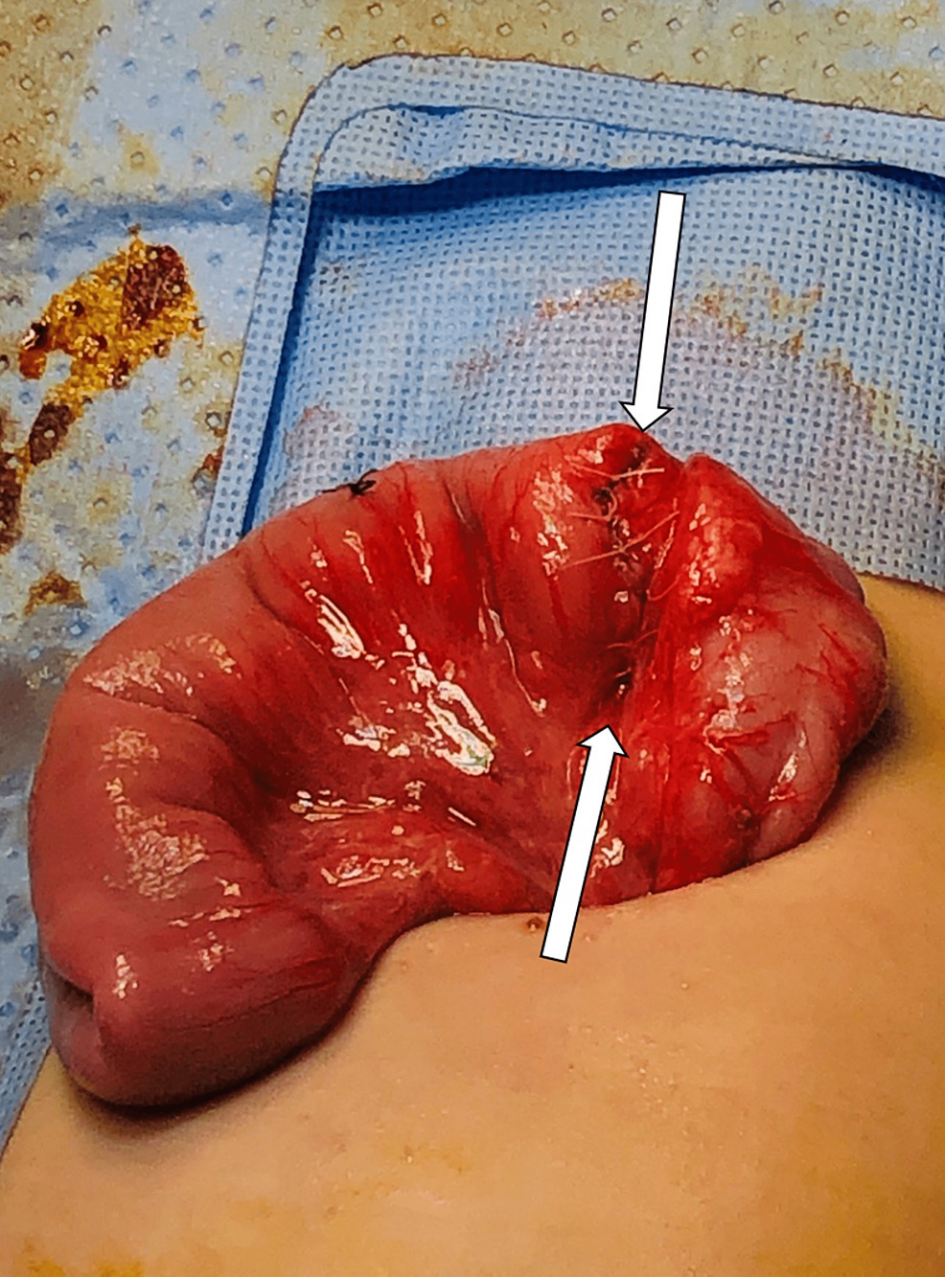
Intraoperative finding Post submucosal enucleation, closure of the resulted seromuscular defect (arrows).

**Figure 12:**
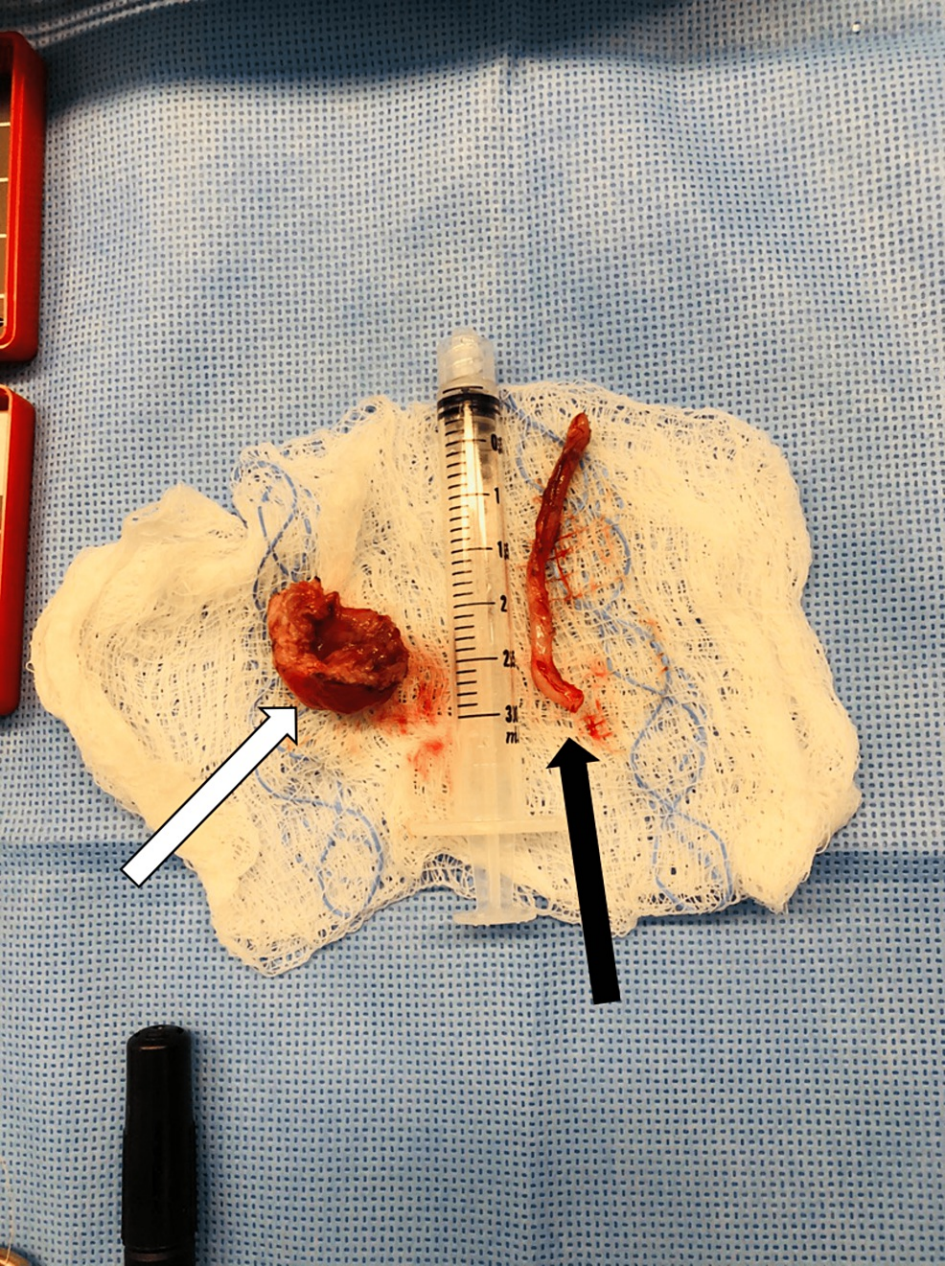
Enucleated cystic lesion The gross appearance of the cystic lesion measured 2 x 1 cm (white arrow) along with the appendix (black arrow).

During the procedure, a duplication cyst was discovered at the lateral outer part of the cecum. The cyst was aspirated, yielding 3-5 ml of pale yellow to clear fluid, which was sent for cytology analysis (Figure 8). The cytology results later confirmed the absence of malignancy, indicating benign glandular epithelium in the fluid. The cyst was then managed through enucleation and closure of the resulting seromuscular defect, along with an appendectomy (as shown in Figures 9-12). The patient had a smooth recovery without any complications. Histopathology reported a benign ectopic gastric tissue negative for malignancy. The baby was discharged in good condition. There were no recurring symptoms during the two-year follow-up period.

## Discussion

The diagnosis of CDC is often challenging due to its presentation as unrelated and atypical symptoms. To differentiate it from intussusception, imaging studies such as abdominal ultrasound or CT are necessary for confirmation. For example, the presence of a hyperechoic wall or a double wall sign on an ultrasound can help identify the cysts. While ultrasonogram is the most commonly used imaging modality for duplication cysts, MRI or CT scans may also be utilized to confirm the diagnosis [12]. Therefore, it is crucial to be knowledgeable about the symptoms and imaging findings of CDCs to avoid any misdiagnosis. In cases where CDCs cause intussusception, the preferred treatment is laparotomy and excision of the cyst [13,14]. However, in certain instances, segmental bowel resection with anastomosis may be necessary [15,16].

Resection and anastomosis is the preferred treatment for CDCs, and this procedure has a generally positive outlook [17]. In instances where CDCs are causing intussusception, laparoscopic-assisted resection is another viable treatment choice [18]. In our case, we elected to convert to an open approach to diagnose and treat that affected part, since it was a suspicious cystic lesion laparoscopically, and imaging modality reported an intussusception as well. Additionally, removing the cyst along with the common ileal wall and performing enterorrhaphy is a safe and effective option for preserving the ileocecal region [19]. Surgical intervention is widely regarded as the best approach for addressing CDCs [18]. Various surgical management techniques have been outlined by researchers [17], including a case report where the cecum was incised on the anti-mesenteric side, the cyst was isolated and then resected while preserving the cecal wall [20], which is considered to be a favorable option.

Our patient underwent surgical extra mucosal enucleation of the cyst, without mucosal perforation. This approach has been documented in the literature, and other surgical techniques and interventions at the lesion site have also been reported.

Early identification is crucial in preventing complications associated with duplication cysts, including perforation, intussusception, volvulus, and malignancy [19]. However, the decision-making process for patients should be personalized, considering their unique attributes, as well as the location and size of the cyst. It is worth noting that the majority of cases demonstrate favorable outcomes following resection [20].

## Conclusions

Our case presents a distinct instance of distal intestinal obstruction resulting from a duplication cyst in the cecum. This case serves as a reminder of the significance of thorough clinical examination and imaging techniques in accurately diagnosing this uncommon condition. Surgical removal is considered the most dependable course of action for CDCs and should be undertaken with the aim of preserving the integrity of the cecal wall, if feasible. Based on the existing evidence, the excision of duplication cysts in children is a secure and efficient treatment option. The surgical approach should be customized to the specific patient and the location of the cyst. Whenever possible, complete removal is advised.
